# Thioredoxin System Protein Expression Is Associated with Poor Clinical Outcome in Adult and Paediatric Gliomas and Medulloblastomas

**DOI:** 10.1007/s12035-020-01928-z

**Published:** 2020-05-16

**Authors:** Anqi Yao, Sarah J. Storr, Khaled Al-hadyan, Ruman Rahman, Stuart Smith, Richard Grundy, Simon Paine, Stewart G. Martin

**Affiliations:** 1 Division of Cancer and Stem Cells, Nottingham Breast Cancer Research Centre, Biodiscovery Institute, University of Nottingham, University Park, Nottingham, NG7 2RD UK; 2 Radiation Biology Section, Biomedical Physics Department, King Faisal Specialist Hospital and Research Centre, Riyadh, Kingdom of Saudi Arabia; 3 Children’s Brain Tumour Research Centre, Biodiscovery Institute, University of Nottingham, University Park, Nottingham, NG7 2RD UK; 4 Department of Neuropathology, Queen’s Medical Centre, Nottingham University Hospitals NHS Trust, Nottingham, NG7 2UH UK; 5 Division of Cancer and Stem Cells, Nottingham Breast Cancer Research Centre, Biodiscovery Institute, University of Nottingham, University Park, Nottingham, NG7 2RD UK

**Keywords:** Thioredoxin, Thioredoxin reductase, Glioblastoma, Low-grade glioma, High-grade glioma, Medulloblastoma

## Abstract

The thioredoxin (Trx) system is an important enzyme family that regulates cellular redox homeostasis. Protein expression of Trx system family members has been assessed in various cancers and linked to various clinicopathological variables, disease progression, treatment response and survival outcomes but information is lacking in brain tumours. Expression of the system was therefore examined, by immunohistochemistry in different brain tumour types, adult and paediatric cases, to determine if expression was of importance to clinical outcome. Trx system proteins were expressed, to variable levels, across all brain tumour types with significant variations in expression between different tumour types/grades/regions. High Trx reductase (TrxR) expression was linked to worse prognosis across all cohorts. High cytoplasmic TrxR expression was significantly associated with adverse overall survival (OS) in adult glioblastoma (*P* = 0.027) and paediatric low-grade glioma (LGG) patients (*P* = 0.012). High expression of nuclear TrxR, cytoplasmic and nuclear Trx and Trx-interacting protein (TxNIP) was associated with improved OS in paediatric LGGs (*P* = 0.031, *P* < 0.001, *P* = 0.044 and *P* = 0.018, respectively). For patients with high-grade gliomas, both high cytoplasmic TrxR and Trx expression were associated with poor OS (*P* = 0.002 and *P* = 0.007, respectively). In medulloblastoma, high expression of cytoplasmic TrxR and Trx and nuclear Trx was associated with worse prognosis (*P* = 0.013, *P* = 0.033 and *P* = 0.007, respectively); with cytoplasmic TrxR and nuclear Trx remaining so in multivariate analysis (*P* = 0.009 and *P* = 0.013, respectively). The consistent finding that high levels of cytoplasmic TrxR are associated with a worse prognosis across all cohorts suggests that TrxR is an important therapeutic target in brain cancers.

**Electronic supplementary material:**

The online version of this article (10.1007/s12035-020-01928-z) contains supplementary material, which is available to authorized users.

## Introduction

Gliomas are the most frequent type of primary brain tumours in both adults and children, with glioblastoma multiforme (GBM) being the most malignant and most common type in adults [1]. Despite advances in diagnosis and treatment, survival times have not significantly improved for malignant glioma patients, especially GBM patients, whose median survival time is only around 15 months from diagnosis with a 5-year survival rate of 6% [2]. Medulloblastoma (MB) is the most frequent primary malignant brain tumour in children [3]. Based on clinical findings and histological subtype, MBs are currently categorised into two groups in clinical practice: average-risk and high-risk disease [4]. Although multimodal treatment strategies are used, 15–20% of the average-risk and 30–40% of the high-risk patients develop recurrences resulting in poor survival outcome [5]. The median survival after relapse is around 10 months [6] and the 5-year survival after relapse is 6% [7]. Collectively, both malignant gliomas and MBs are highly invasive tumours, with recurrence after treatment almost inevitable, resulting in extremely poor prognosis. There is, therefore, a pressing need to identify reliable and robust prognostic biomarkers to better stratify patients in order to improve their survival and to identify novel targets for development of therapeutics.

Redox homeostasis is often disrupted in cancer cells due to increased oxidative stress caused by the accelerated proliferation, high metabolic rate and persistent growth-promoting signalling pathways of tumours [8]. To maintain the redox balance, antioxidant systems such as the thioredoxin (Trx) system are often deregulated/overexpressed in aggressive tumours to counteract the increased oxidative stress [9]. The Trx system is a key antioxidant pathway in defence against oxidative stress and comprises Trx reductase (TrxR), its ubiquitous substrate Trx and the endogenous inhibitor of this system - Trx-interacting protein (TxNIP) [10]. TrxR reduces oxidised disulphide-containing Trx back to its biologically active dithiol form in a NADPH-dependent manner and the reduced Trx, in turn, reduces oxidised cysteine groups on downstream proteins [11]. TxNIP inhibits Trx activity and its interactions with downstream factors by directly binding to the catalytic centre of Trx [12]. The Trx system plays a key role in maintaining redox-regulated cellular functions including transcription, DNA damage recognition and repair, proliferation and apoptosis [11, 13].

The Trx system proteins are deregulated/overexpressed in a number of different cancers with the level of expression often associated with tumour aggressiveness, metastasis, prognosis and treatment responses [9, 14–18]. There is very little information regarding the association of this system with clinicopathological factors and prognosis in brain tumours, especially paediatric gliomas and medulloblastomas. Jarvela et al. indicated that increased Trx expression was associated with higher tumour grade and poor prognosis in oligodendrogliomas [19]. Haapasalo et al. found a positive association between the expression of Trx/TrxR and tumour grade in astrocytomas with only high Trx expression significantly associated with poor prognosis [20]. The levels of TrxR in GBM patients were shown to be higher than in normal brain tissue, indicating that TrxR may be related to the progression of GBM [21]. In a recent study, low TxNIP expression in glioma was associated with higher histological grade and shorter patient survival [22].

Although the role of Trx system has been studied in malignant gliomas, few studies have assessed the prognostic value of this Trx system as a whole across a variety of brain tumour types and there have been no studies conducted in paediatric patients. The aim of the current study was, therefore, to use immunohistochemical approaches to assess the expression of all three members of the Trx system in four different brain tumour cohorts, to evaluate their associations with clinicopathological and survival criteria and, additionally, to compare levels of expression across different tumour regions/types/grades.

## Material and Methods

### Clinical Samples

This study is reported in accordance with REMARK (reporting recommendations for tumour marker prognostic studies) criteria [23]. Ethical approval was granted by the Local Regional Ethics Committee in Nottingham, under the title ‘Comparative molecular analysis of childhood brain tumours’ (11/EM/0076; R&I 14CS005). Four independent cohorts were used in this study: adult GBM, paediatric low-grade glioma (LGG) (WHO grade I and II), paediatric high-grade glioma (HGG) (WHO grade III and IV) and MB cohorts. A total of 395 primary brain tumour patients were diagnosed and treated at various hospitals across England (mainly Nottingham and Birmingham) between 1970 and 2015. Exclusion of referral, miscoded and recurrent cases resulted in 302 cases with available clinical information. The clinicopathological variables of the cohorts are listed in Supplementary Table 1-3. Overall survival time was calculated from the date of original diagnosis to death or from date of original diagnosis to last date known to be alive for those censored.

The 18 cases in the adult GBM cohort were treated at Nottingham University Hospitals between 2013 and 2015. The median age for this cohort was 59 years (ranging from 26 to 73). The median follow-up time was 15 months (ranging 1–42). Patients were managed under a uniform protocol, where all underwent complete (14/18) or partial resection (4/18), as decided by disease characteristics. The majority of the patients (16/18) completed 60 Gray (2 Gray per fraction over 6 weeks) radiotherapy and adjuvant temozolomide treatment whilst the remaining 2 patients only received radiotherapy.

The 126 paediatric patients in the LGG cohort were treated at various hospitals in England between 1970 and 2003. The median age for this cohort was 6 years (ranging from 3 months to 16 years) and 81% of patients had grade I disease (92/114); where patient number did not total 126, information was not available for the remaining patients. The median follow-up time was 168 months (ranging 0–416). Unfortunately, surgical and adjuvant treatment data were not available for this cohort.

The paediatric HGG cohort included 137 patients who were diagnosed at various hospitals in England between 1982 and 2007. The median follow-up time was 12 months (ranging 0–206). 26.7% of patients had grade III tumours (35/131) and 73.3% of patients had grade IV tumours (96/131). 15.8% of patients had complete tumour resection (16/101), 43.6% of patients had partial resection (44/101) and 40.6% of patients had biopsies (41/101). Where patient number did not total 137 information was not available for the remaining patients. The adjuvant treatment data for this cohort were also not available.

In the MB cohort, 114 patients were diagnosed and treated at Birmingham and Nottingham University Hospitals between 1985 and 2012. The median follow-up time was 53 months (ranging 1-249). The median age of the patients was 7 years (ranging from 9 months to 19 years). According to conventional histological classification, 61.3% of patients were classified as classic MB (57/93), 16.1% as desmoplastic MB (15/93), 17.2% as large cell/anaplastic MB (16/93), 1.1% as MB with extensive nodularity (1/93) and 4.3% as MB with myogenic differentiation (4/93). The metastatic stage of MBs was determined according to the Modified Chang Staging System [24], where the extent of metastasis is subdivided into M0 (no metastasis), M1 (presence of tumours in the cerebrospinal fluid), M2 (gross nodular seeding present intracranially beyond the primary tumour site), M3 (metastasis in spinal subarachnoid space) and M4 (metastases outside the cerebrospinal axis). Among the 92 patients with available metastatic information, 60.9% of patients were M0 stage (56/92), 6.5% of patients were M1 stage (6/92), 10.9% of patients were M2 stage (10/92), 18.5% of patients were M3 stage (17/92) and 3.3% of patients were M4 stage (3/92). As with the paediatric LGG and HGG cohorts, the treatment details were also not available for this cohort.

### Tissue Microarray and Immunohistochemistry

Protein expression was assessed using freshly cut 4-μm sections from tissue microarrays (TMAs). TMAs were kindly provided to the current authors by Prof. Richard Grundy and Mr. Stuart Smith from the Children’s Brain Tumour Research Centre, the University of Nottingham, UK. For paediatric LGG, HGG and MB TMAs, two/three 0.6-mm tissue cores were used for each patient. Adult GBM TMAs were constructed as described previously [25]. In short, triplicate cores were collected from each of three regions (inner tumour core, rim and invasive margin) per tumour specimen.

Following optimisations immunohistochemistry (IHC) was performed as previously described using a Novolink Polymer Detection Kit (Leica) according to the manufacturers’ instructions [26]. Briefly, slides were dewaxed in xylene and rehydrated in industrial methylated spirit (IMS), followed by antigen retrieval in 0.01 M sodium citrate buffer (pH 6.0) in a microwave for 20 min, with 10 min at 750 W and 10 min at 450 W. Tissue was treated with peroxidase block for 5 min, washed with Tris-buffered saline (TBS) and then treated with protein block solution for another 5 min at room temperature. Primary antibodies against Trx (1:2000, Abcam), TrxR (1:150, Abcam) and TxNIP (1:1000, Abcam) were incubated on tissue overnight at 4 °C, with antibody specificity initially confirmed by Western blotting (Supplementary Fig. 1). Following antibody incubation, tissues were washed with TBS and incubated with post primary solution, then washed and incubated with Novolink polymer solution. Immunohistochemical reactions were visualised using 3, 3′-diaminobenzidine and counterstained with haematoxylin. Slides were then dehydrated in IMS, fixed in xylene before mounting with DPX. Controls were included with each run: breast tumour composite sections, comprised of grade 1 and 2 early-stage invasive tumours, were included as positive controls, with negative controls omitting primary antibody.

### Assessment of Expression

Staining was assessed at × 200 magnification following high-resolution scanning (Nanozoomer Digital Pathology Scanner, Hamamatsu Photonics). Assessment was conducted with involvement and training provided by a consultant neuropathologist (SP). Cytoplasmic staining was semi-quantitatively assessed using an immunohistochemical *H*-score [27], where staining intensity was assessed as none (0), weak (1), medium (2), or strong (3) over the percentage area of each staining intensity. Nuclear staining was assessed as the percentage of nuclei with any intensity of staining. 30% of cores for each protein were examined by a second independent assessor blinded to clinical outcome and the primary assessor’s scores. Good concordance was demonstrated between scorers (single measure intraclass correlation coefficients were above 0.7 for all markers assessed across all brain tumour cohorts) (Supplementary Table 4). Unbiased cut-points, for stratification, were obtained based on overall survival using X-tile software [28].

### Statistical Analysis

Data analysis was performed using SPSS 24.0 software. The relationships between categorised protein expression and clinicopathological variables were examined using Pearson’s *χ*^2^ test of association or Fisher’s exact test if a cell count was less than 5 in a 2 × 2 table. Kaplan–Meier survival curves were plotted with significance determined using the Log-rank test. The Cox proportional hazards regression model was used in multivariate survival analysis. The differences in protein expression between different tumour types/grades/regions were determined using one-way ANOVA or non-parametric Kruskal-Wallis test when the homogeneity of variance was violated. Multiple comparisons between two groups were conducted using a pairwise post hoc *t* test or Mann–Whitney *U* test when the data were not normally distributed. *P* values < 0.05 were considered statistically significant.

## Results

### Expression Pattern of Trx System in Brain Tumours

**Fig. 1:**
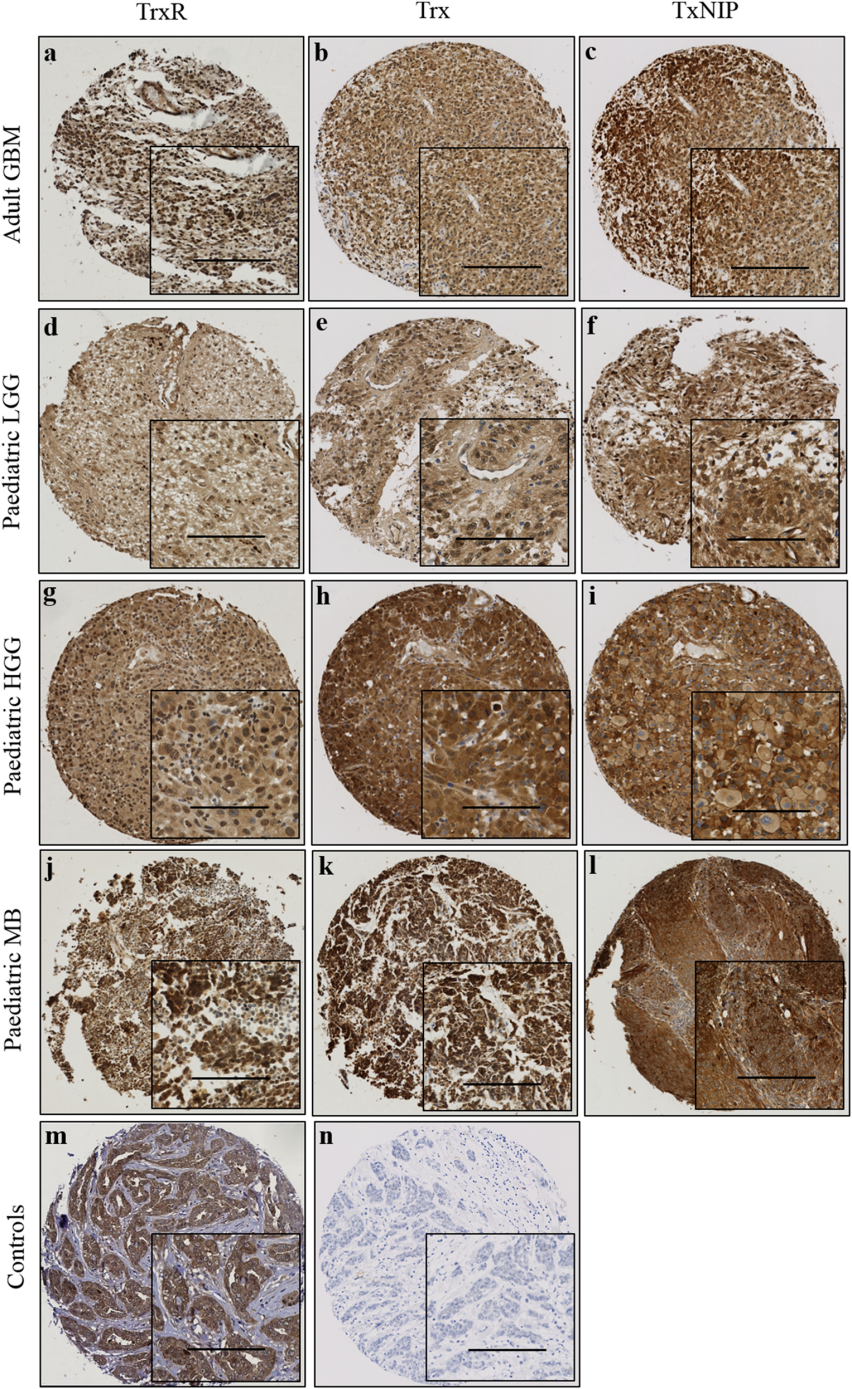
Representative photomicrographs of Trx system expression in different brain tumour types. Examples of TrxR (left panel), Trx (middle panel) and TxNIP (right panel) staining in **a–c** adult glioblastoma; **d–f** paediatric low-grade glioma; **g–i** paediatric high-grade glioma; **j–l** paediatric medulloblastoma; **m** representative positive control of TrxR on breast cancer tissue; **n** representative negative control with omission of the primary antibody on breast cancer tissue. Images were taken at × 10 magnification with × 20 magnification inset panel. Scale bar represents 100 μm

TrxR, Trx and TxNIP expression displayed a mixture of diffuse and granular cytoplasmic staining in all types of brain tumours. Some diffuse nuclear staining was also observed for TrxR and Trx. Representative staining patterns are shown in Fig. 1. Heterogeneous staining was shown between, as well as within, certain tumour cores for all markers, varying from weak to intense. Occasional stromal and endothelial cell staining was also observed; however, this was not scored as part of this study. The median scores and ranges of TrxR, Trx and TxNIP expression for each tumour cohort are shown in Supplementary Table 5 with X-tile cut-off points listed in Supplementary Table 6. TMA cores with insufficient tumour cells (< 15%) were not scored or included in the analysis.

### Relationship of Trx System Expression with Clinicopathological Variables and Clinical Outcome in the Adult GBM Cohort

In the adult GBM cohort, the relationships between Trx system protein expression and clinicopathological variables were independently assessed by tumour region (i.e. core, rim and invasive margin). In core samples, high nuclear TrxR expression was associated with tumours located at temporal lobe (*χ*^2^ = 9.370, *df* = 3, *P* = 0.008); in samples taken from the invasive margin, high cytoplasmic TrxR expression was also linked with male patients (*χ*^2^ = 8.571, *df* = 1, *P* = 0.015) (Supplementary Table 7). No significant associations between Trx or TxNIP expression and clinicopathological variables were observed in any tumour regions (data not shown).

**Fig. 2:**
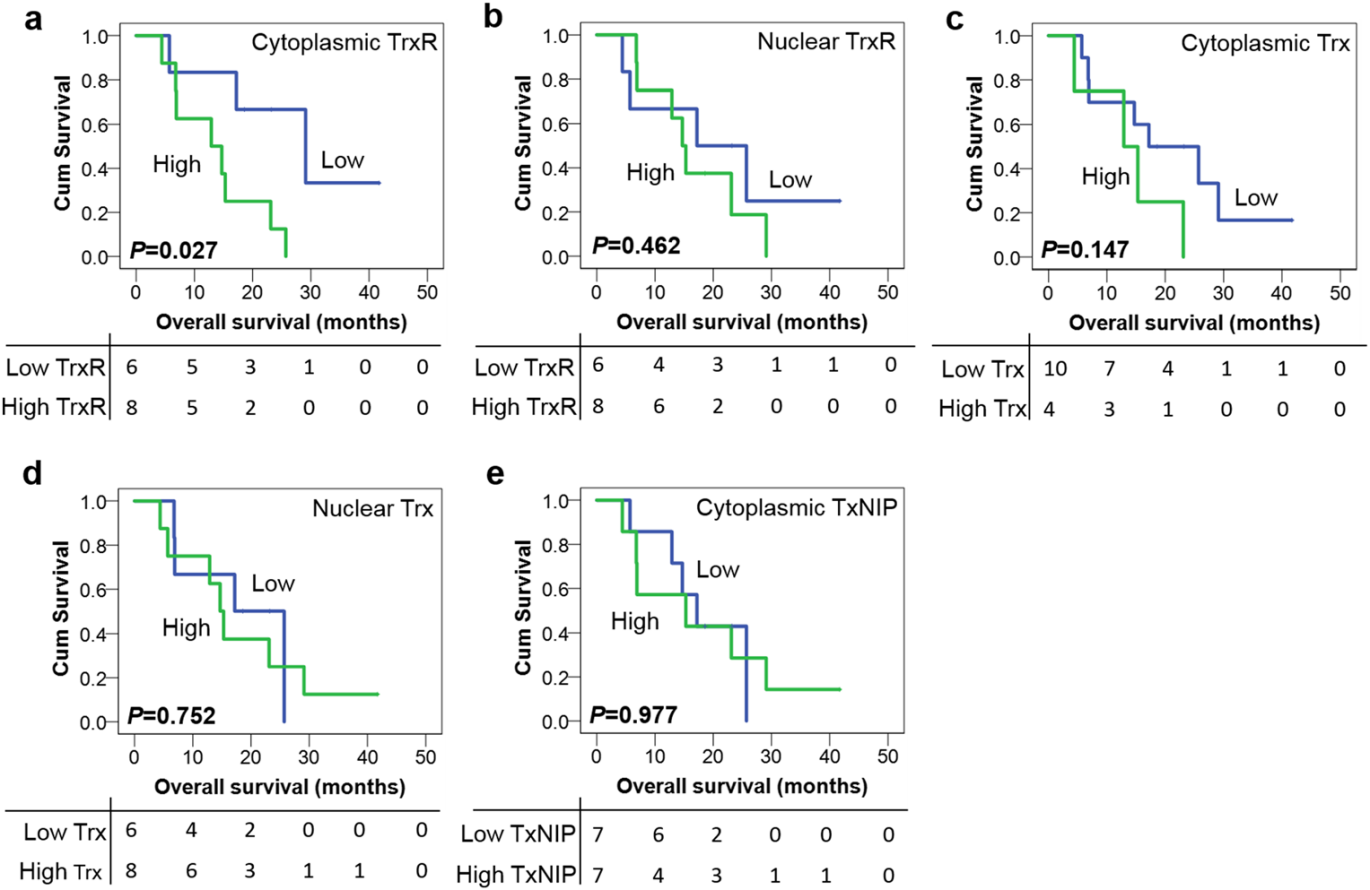
Kaplan–Meier overall survival curves in the adult GBM cohort (rim). **a** High expression of cytoplasmic TrxR is associated with poor overall survival (*P* = 0.027). No significant associations between nuclear TrxR (**b**), cytoplasmic/nuclear Trx (**c/d**) or TxNIP (**e**) expression and overall survival are observed (all *P* values > 0.05). Curves show low (blue line) and high protein expression (green line) with significance determined using the log-rank test. The numbers below the Kaplan–Meier curves are the number of patients at risk at the specified month

In rim samples high cytoplasmic TrxR expression was significantly associated with adverse overall survival (*P* = 0.027) whereas nuclear TrxR expression was not (*P* = 0.462) (Fig. 2 a and b). The expression of cytoplasmic/nuclear Trx and TxNIP was also not associated with overall survival (*P* = 0.147, 0.752 and 0.977, respectively) (Fig. 2c–e). No associations were observed between TrxR, Trx and TxNIP expression and overall survival in either core or invasive samples (data not shown). In addition, none of the traditional prognostic variables including patient age, tumour site, resection status and IDH-1 status was associated with patient survival (with individual Kaplan–Meier statistics of *P* = 0.396, *P* = 0.484, *P* = 0.855 and *P* = 0.643 respectively). Therefore, multivariate analysis was not performed to determine whether cytoplasmic TrxR had independent prognostic value in rim samples.

### Relationship of Trx System Expression with Clinicopathological Variables and Clinical Outcome in the Paediatric LGG Cohort

In the paediatric LGGs, high cytoplasmic TrxR was associated with supratentorial tumours (*χ*^2^ = 10.384, *df* = 1, *P* = 0.002) and the presence of tumour recurrence (*χ*^2^ = 10.231, *df* = 1, *P* = 0.004), whereas high nuclear TrxR expression was associated with the absence of tumour recurrence (*χ*^2^ = 9.850, *df* = 1, *P* = 0.001). High expression of both cytoplasmic Trx and TxNIP was also associated with the absence of tumour recurrence (*χ*^2^ = 5.663, *df* = 1, *P* = 0.029 and *χ*^2^ = 6.147, *df* = 1, *P* = 0.013, respectively). The expression of nuclear Trx was not significantly associated with any of the clinicopathological variables (Supplementary Table 8 and Table 9).

**Fig. 3:**
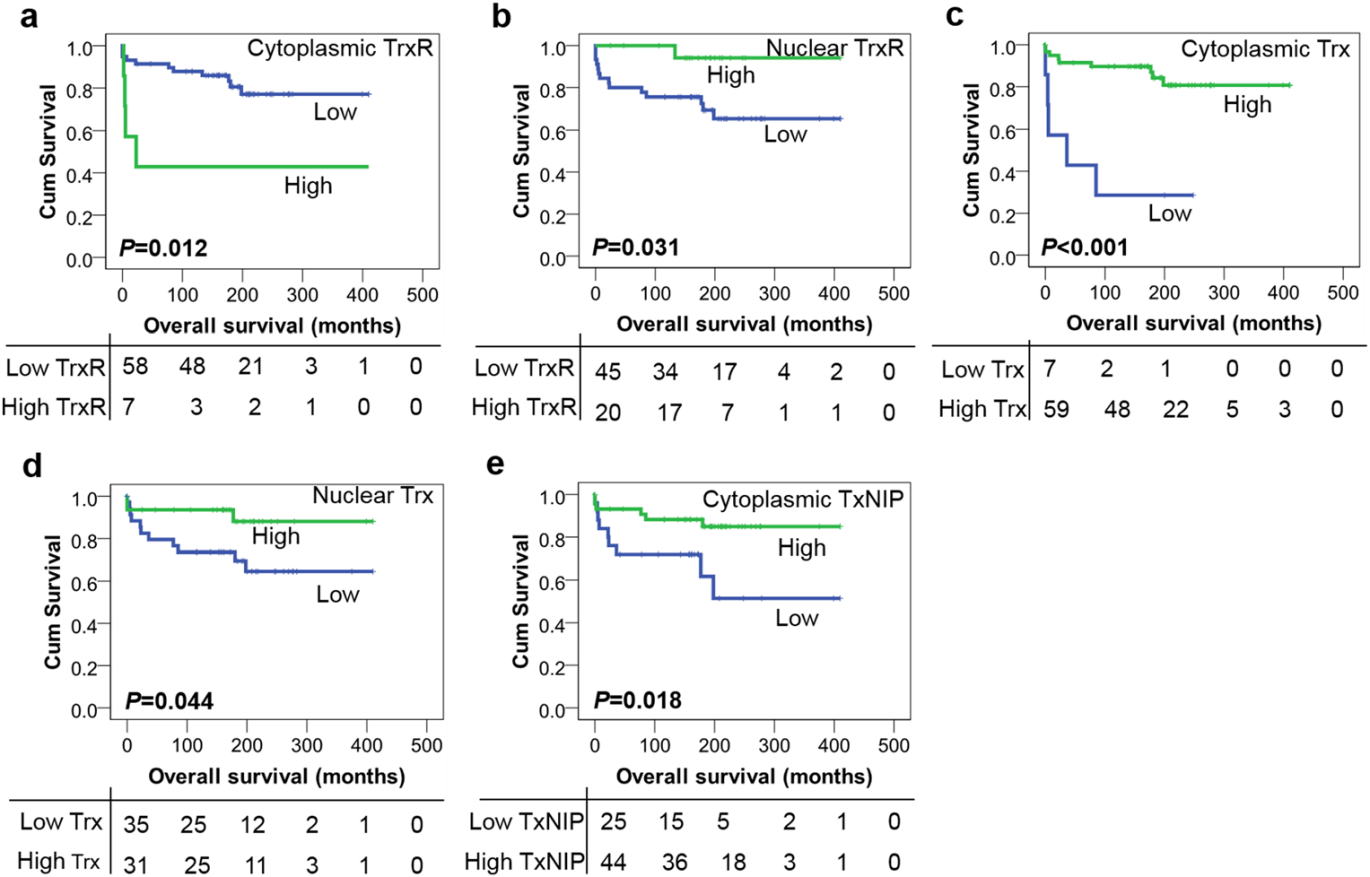
Kaplan–Meier overall survival curves in the paediatric LGG cohort. **a** High expression of cytoplasmic TrxR is associated with adverse overall survival (*P* = 0.012) whilst high expression of nuclear TrxR (**b**) is correlated with better overall survival (*P* = 0.031). High expression of cytoplasmic (**c**) or nuclear (**d**) Trx is associated with improved overall survival (*P* < 0.001 and *P* = 0.044, respectively). **e** High expression of TxNIP is also associated with better overall survival (*P* = 0.018). Curves show low (blue line) and high protein expression (green line) with significance determined using the log-rank test. The numbers below the Kaplan–Meier curve are the number of patients at risk at the specified month

Kaplan–Meier survival analysis showed that high expression of cytoplasmic TrxR was significantly associated with poor overall survival (*P* = 0.012) (Fig. 3a) whereas high TrxR expression in the nucleus was associated with improved overall survival (*P* = 0.031) (Fig. 3b). High expression of Trx within both cytoplasm and nucleus was significantly associated with improved overall survival (*P* < 0.001 and *P* = 0.044, respectively) (Fig. 3c and d). In addition, high TxNIP expression was also associated with improved overall survival (*P* = 0.018) (Fig. 3e). The multivariate analysis was also not performed in this cohort as none of the potential confounding factors were significantly associated with patient survival including tumour grade, tumour site, patient age and gender (with individual Kaplan–Meier statistics of *P* = 0.058, *P* = 0.284, *P* = 0.613 and *P* = 0.939, respectively).

### Relationship of Trx System Expression with Clinicopathological Variables and Clinical Outcome in the Paediatric HGG Cohort

In the paediatric HGGs, anaplastic oligodendroglioma was significantly associated with high cytoplasmic Trx expression whilst GBM with low expression (*χ*^2^ = 9.785, *df* = 2, *P* = 0.008). High cytoplasmic TxNIP expression was linked with GBM (*χ*^2^ = 9.362, *df* = 2, *P* = 0.009), supratentorial tumours (*χ*^2^ = 5.314, *df* = 2, P = 0.021) and higher tumour grade (*χ*^2^ = 7.923, *df* = 1, *P* = 0.005) (Supplementary Table 10). No associations between both cytoplasmic and nuclear TrxR expression and clinicopathological variables were observed (data not shown).

**Fig. 4:**
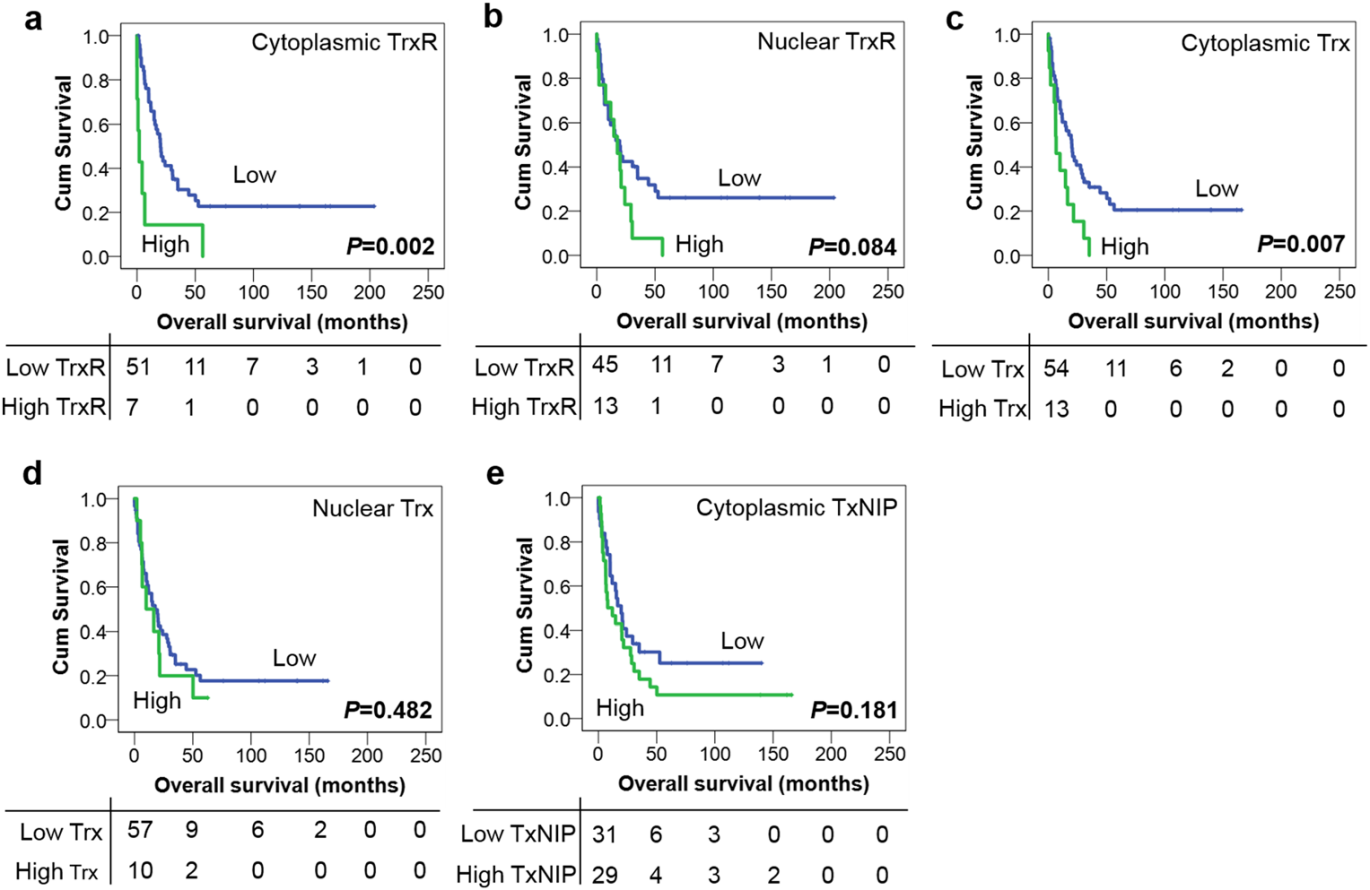
Kaplan–Meier overall survival curves in the paediatric HGG cohort. High expression of cytoplasmic TrxR (**a**) and Trx (**c**) is associated with adverse overall survival (*P* = 0.002 and *P* = 0.007, respectively). No significant associations are found between the expression of nuclear TrxR (**b**), nuclear Trx (**d**), or TxNIP (**e**) and overall survival (*P* = 0.084, 0.482 and 0.181, respectively). Curves show low (blue line) and high protein expression (green line) with significance determined using the log-rank test. The numbers below the Kaplan–Meier curves are the number of patients at risk at the specified month

Univariate survival analysis showed that high expression of both cytoplasmic TrxR and Trx was significantly associated with adverse overall survival (*P* = 0.002 and *P* = 0.007, respectively) (Fig. 4 a and c), whilst expression of both TrxR and Trx in the nucleus was not significantly associated with overall survival (*P* = 0.084 and *P* = 0.482 respectively) (Fig. 4 b and d). No association was detected between TxNIP expression and overall survival (*P* = 0.181) (Fig. 4e). Again, multivariate analysis could not be conducted as none of the potential confounding factors, namely age at diagnosis, gender, histological subtypes, tumour grade, tumour site and extent of surgery, were found to be significantly associated with survival in univariate analysis (with individual Kaplan–Meier statistics of *P* = 0.224, *P* = 0.949, *P* = 0.211, *P* = 0.085, *P* = 0.688 and *P* = 0.494, respectively).

### Relationship of Trx System Expression with Clinicopathological Variables and Clinical Outcome in the Paediatric MB Cohort

In MBs, high expression of both cytoplasmic and nuclear Trx was associated with the presence of tumour recurrence (*χ*^2^ = 4.655, *df* = 1, *P* = 0.031 and *χ*^2^ = 5.851, *df* = 1, *P* = 0.016, respectively). High nuclear Trx expression was also linked with extensive nodularity subtype whilst low expression with classic subtype (*χ*^2^ = 9.941, *df* = 4, *P* = 0.041) (Supplementary Table 11). No other significant associations were found between protein expression and clinicopathological variables (data not shown).

**Fig. 5:**
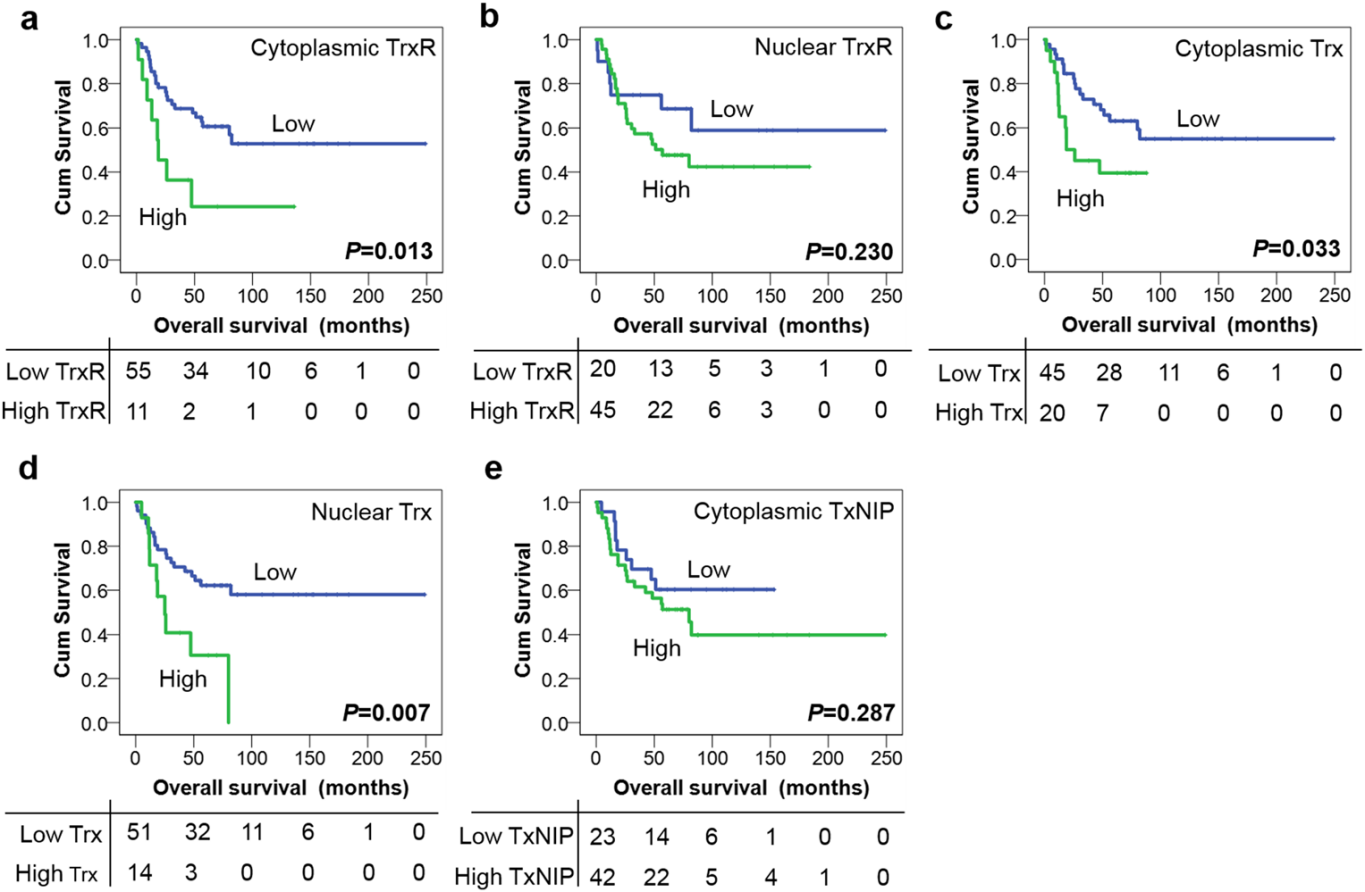
Kaplan–Meier overall survival curves in the MB cohort. High expression of cytoplasmic TrxR (**a**), cytoplasmic Trx (**c**) and nuclear Trx (**d**) is associated with adverse overall survival (*P* = 0.013, *P* = 0.033 and *P* = 0.007, respectively). No significance is observed between nuclear TrxR (**b**) or TxNIP (**e**) expression and overall survival (*P* = 0.230 and *P* = 0.287, respectively). Curves show low (blue line) and high protein expression (green line) with significance determined using the log-rank test. The numbers below the Kaplan–Meier curve are the number of patients at risk at the specified month

As with the other cohorts patients with high cytoplasmic TrxR expression had significantly shorter overall survival compared with low expression (*P* = 0.013) (Fig. 5a). High Trx expression within cytoplasm and nucleus were both associated with adverse overall survival (*P* = 0.033 and *P* = 0.007, respectively) (Fig. 5 c and d). No significance was observed between nuclear TrxR or TxNIP expression and overall survival (*P* = 0.230 and *P* = 0.287, respectively) (Fig. 5 b and e). In multivariate Cox regression analysis, cytoplasmic TrxR and nuclear Trx expression both remained significantly associated (*P* = 0.009 and 0.013, respectively) with overall survival when including the potential confounding factors of age, extent of surgery and metastatic status in the analysis (with individual Kaplan–Meier statistics of *P* = 0.001, *P* = 0.038 and *P* < 0.001, respectively), whereas cytoplasmic Trx expression was not independently associated with survival (*P* = 0.206) (Table 1).

**Table 1:** Multivariate Cox proportional hazards analysis for predictors of overall survival in medulloblastoma patients

Variables	*P* value	Exp (B)	95% CI for Exp(B)	
			Lower	Upper
Cytoplasmic TrxR expression	*0.009*	4.850	1.487	15.822
Age (3 years)	*0.004*	0.190	0.062	0.585
Extent of surgery	0.397	0.643	0.232	1.785
Metastatic status	*0.000*	6.354	2.266	17.816
Cytoplasmic Trx expression	0.206	1.895	0.703	5.107
Age (3 years)	*0.007*	0.221	0.074	0.662
Extent of surgery	0.549	0.716	0.240	2.132
Metastatic status	*0.003*	5.640	1.812	17.555
Nuclear Trx expression	*0.013*	5.386	1.418	20.451
Age (3 years)	*0.003*	0.188	0.062	0.572
Extent of surgery	0.097	0.311	0.079	1.233
Metastatic status	*0.011*	4.033	1.375	11.827

Significant *P* values are indicated in italic. Exp(B) is used to denote hazard ratio and 95% CI is used to denote 95% confidence interval

### Comparison of Protein Expression Between Different Regions in Adult GBM

**Fig. 6:**
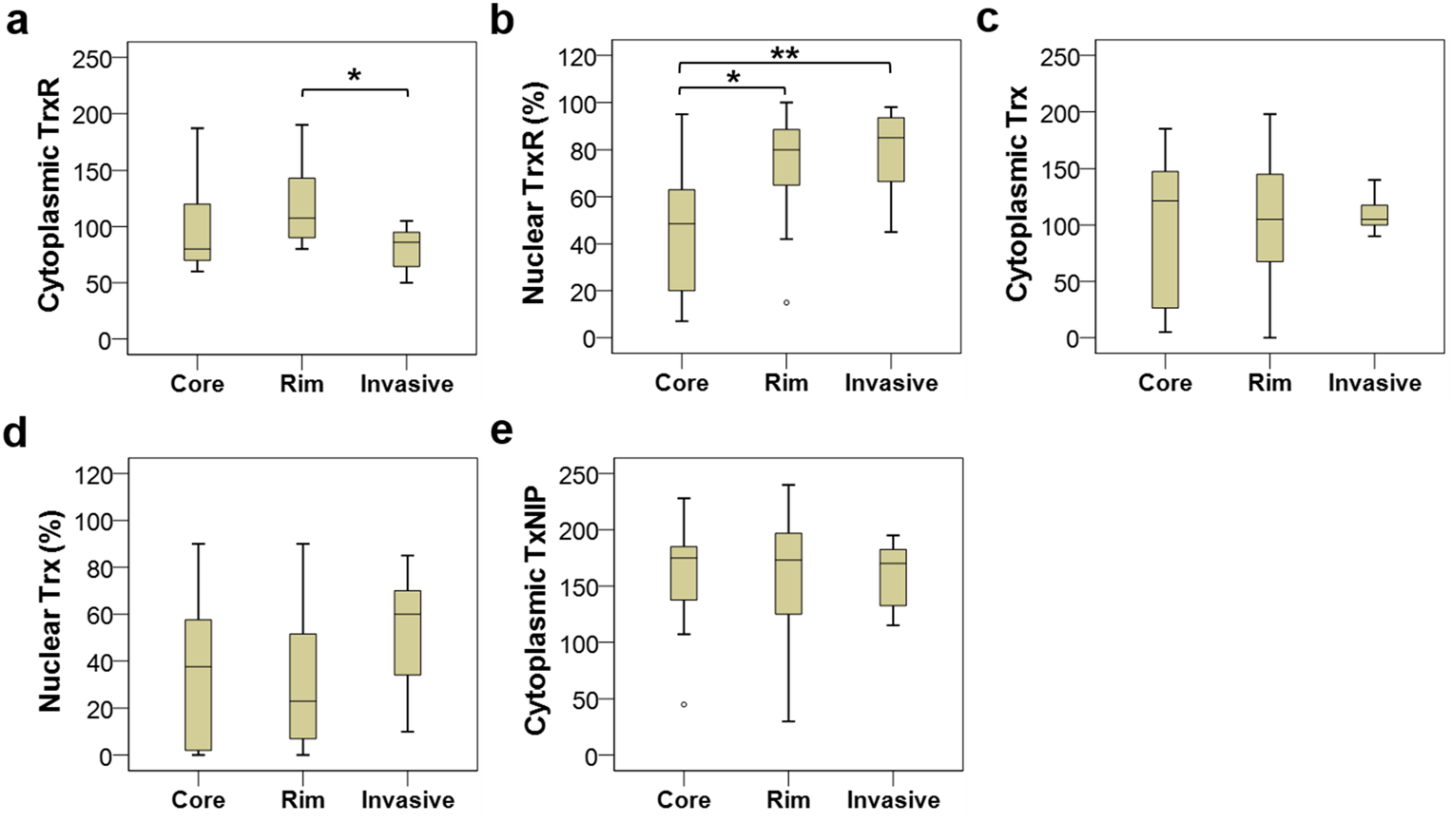
Box plots of protein expression in different regions of adult GBM tumours. (**a**) A significantly higher expression of cytoplasmic TrxR is noted in the rim samples compared with the invasive samples (*P* = 0.036 on post hoc *t* test). (**b**) Significant differences in nuclear TrxR expression were observed between core and rim regions (*P* = 0.019) and between core and invasive regions (*P* = 0.004) but not between rim and invasive regions (*P* = 0.724). No statistically significant variations are detected between areas for cytoplasmic or nuclear Trx (**c** and **d**, respectively) or TxNIP expression (**e**). *P < 0.05, **P < 0.01

As shown in Fig. 6a, the levels of cytoplasmic TrxR expression significantly differed between the core, rim and invasive regions of adult GBM tumours (*P* = 0.043). On a post hoc *t* test, there was a significantly higher level of cytoplasmic TrxR in the rim region than the invasive region (*P* = 0.036), but there were no significant differences between core and rim (*P* = 0.713) or core and invasive regions (*P* = 0.190). A significant difference in nuclear TrxR expression was also observed between these three intra-tumour regions (*P* = 0.003). On post hoc *t* test, there was a significant difference between core and rim samples (*P* = 0.019), and between core and invasive samples (*P* = 0.004), but not between the rim and invasive samples (*P* = 0.724) (Fig. 6b). The expression levels of cytoplasmic/nuclear Trx and TxNIP were also compared between different GBM areas, but no statistically significant variations were detected (*P* = 0.987, 0.442 and 0.944, respectively) (Fig. 6c–e).

### Comparison of Protein Expression Between Different Tumour Types

**Fig. 7:**
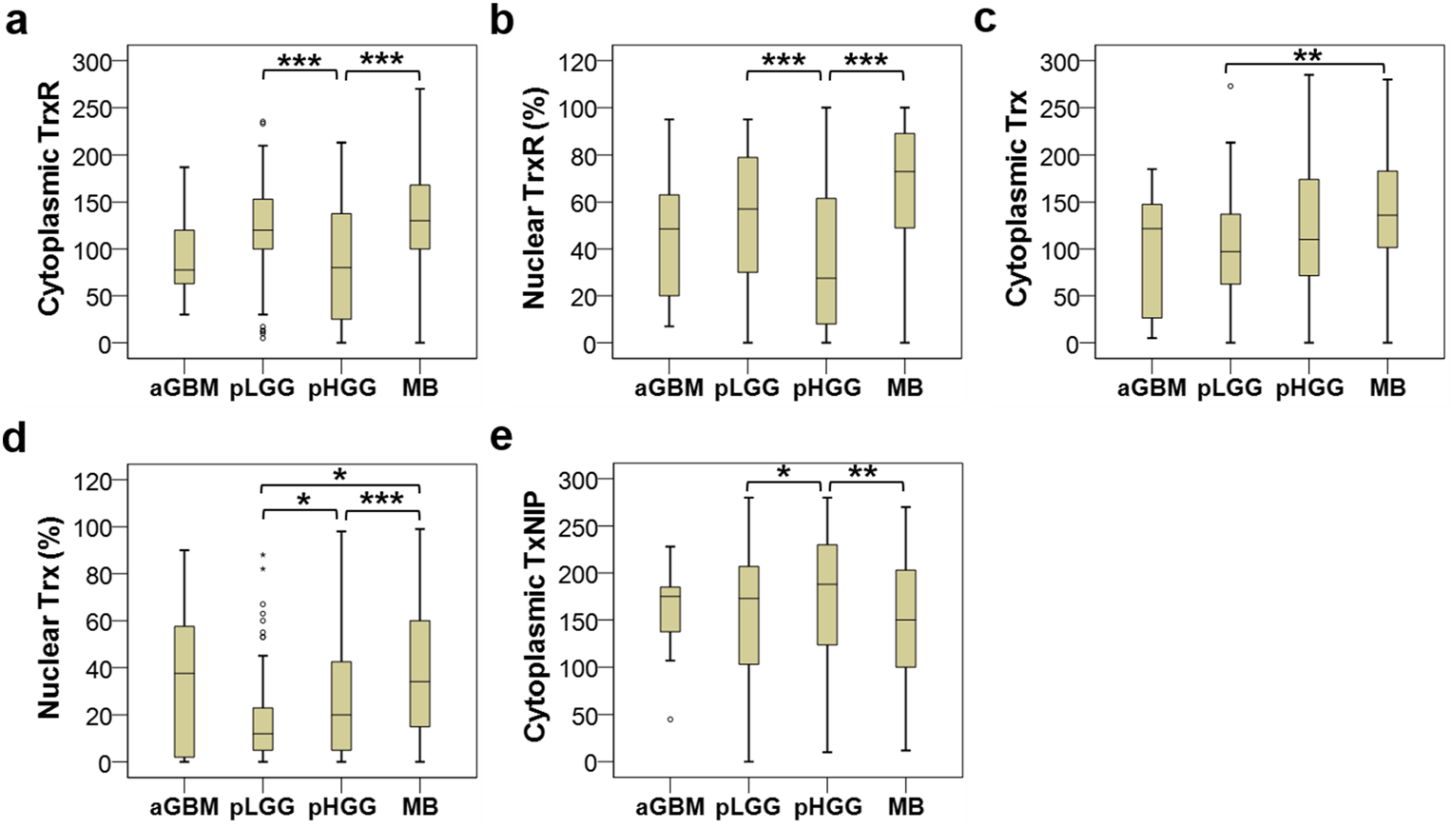
Box plots of protein expression in different brain tumour types. The expression of both cytoplasmic (**a**) and nuclear (**b**) TrxR is significantly lower in pHGG than pLGG and MB (*P* < 0.001). Cytoplasmic Trx (**c**) expression is significantly higher in MB compared with pLGG (*P* = 0.004), and nuclear Trx (**d**) expression significantly differs between pLGG, pHGG and MB (*P* < 0.001), with pLGG having the lowest level and MB having the highest level. **e** A significantly higher expression of TxNIP is noted in pHGG compared with pLGG and MB (*P* = 0.017). *P < 0.05, **P < 0.01, ***P < 0.001

There was a significant difference in cytoplasmic TrxR expression between adult GBMs, paediatric LGGs, paediatric HGGs and MBs (*P* < 0.001) (Fig. 7a). From the post hoc *t* test, the significant differences were observed between paediatric LGGs and HGGs (*P* < 0.001), and between paediatric HGGs and MBs (*P* < 0.001), whereas no significant differences were detected between adult GBM and any of the other three tumour types. Statistically significant variations of nuclear TrxR expression were also observed between these four brain tumour types (*P* < 0.001), with significant differences detected between paediatric LGGs and HGGs (*P* < 0.001) and between paediatric HGGs and MBs (*P* < 0.001) (Fig. 7b).

There was a significant difference in cytoplasmic Trx expression between different types of brain tumours (*P* = 0.003) and the difference between paediatric LGGs and MBs was the only pair that achieved statistical significance (*P* = 0.004) (Fig. 7c). Significant differences in nuclear Trx expression were also found between different brain tumour types (*P* < 0.001) (Fig. 7d). MBs showed significantly higher levels of nuclear Trx expression, relative to both the paediatric LGGs (*P* = 0.011) and HGGs (*P* < 0.001); and paediatric HGGs displayed significantly greater levels of such expression than the paediatric LGGs (*P* = 0.027).

The levels of TxNIP expression also differed between different tumour types (*P* = 0.017). Median TxNIP expression was highest for the paediatric HGGs (188, range 10–280), followed by adult GBMs (175, range 45–228) and paediatric LGGs (173, range 0–280) and lowest for MBs (150, range 12–270) (Fig. 7e). There were significant differences between paediatric LGGs and HGGs (*P* = 0.022), and between paediatric HGGs and MBs (*P* = 0.004).

### Comparison of Protein Expression Between Different Tumour Grades in Paediatric Gliomas

In total, there were 159 valid cases of paediatric gliomas including 82 grade I, 25 grade III and 52 grade IV gliomas. Unfortunately, the grade II gliomas were not included in this analysis, as scoring was not able to be conducted due to missing cores or insufficient tumour tissues. As shown in Supplementary Fig. 2a, the expression of cytoplasmic TrxR was significantly higher in grade I gliomas than both grade III (*P* = 0.010) and grade IV gliomas (*P* = 0.001). Like cytoplasmic TrxR expression, grade I gliomas also showed the highest level of nuclear TrxR expression whilst the grade III gliomas had the lowest level (Supplementary Fig. 2b). A significant difference in nuclear TrxR expression was observed between grade I and grade IV gliomas (*P* = 0.001), but not between grade I and III (*P* = 0.085) or between grade III and IV gliomas (*P* = 0.974).

The expression levels of cytoplasmic Trx were nearly equal between grade I, III and IV gliomas with median values of *H*-scores ranging from 92 to 101 (*P* = 0.876) (Supplementary Fig. 2c). Nuclear Trx expression was elevated in both grade III and grade IV gliomas compared with grade I’s but this did not achieve statistical significance (*P* = 0.448) (Supplementary Fig. 2d). As shown in Supplementary Fig. 2e, TxNIP expression increased as pathological grades increased, with the expression being significantly higher in grade IV than in grade I gliomas (*P* = 0.001), but no difference was noted between grade I and III (*P* = 0.556) or between grade III and IV gliomas (*P* = 0.052).

## Discussion

The current study investigated the expression of all three Trx system proteins in four independent brain tumour cohorts (i.e. adult GBM, paediatric LGG, paediatric HGG and MB); and whether any associations existed between their expression and patient prognosis or with clinicopathological variables. The difference in the levels of Trx system protein expression between different tumour regions/types/grades was also investigated. This is the first study to report upon the expression of all three members of Trx system proteins together in such a broad variety of brain tumour types and to evaluate their prognostic values.

The Trx system is a key member of the cellular systems responsible for regulating redox homeostasis. Protein overexpression of Trx system members has been demonstrated in many human cancers with expression often associated with increased tumour progression and worse patient prognosis. Although there are a few studies that have investigated Trx system protein expression in GBMs, none have assessed prognostic importance with this being the first study to examine expression of all three members of the system together in adult GBM patients and to evaluate their prognostic significance. Current survival data demonstrate that high cytoplasmic TrxR expression is significantly associated with adverse overall survival in adult GBMs (rim area) (*P* = 0.027). In a study of 27 GBM cases, Kemerdere et al. [21] reported that serum and tissue levels of TrxR were increased in GBM patients, but this study did not assess associations with patient survival. A study investigating 40 GBM patients showed significantly higher TrxR expression in primary GBMs with TrxR expression highly correlating with the Ki-67 index, but the study also did not assess for associations with patient survival [29]. A further study assessed TrxR in 20 GBM patients, showing higher TrxR expression in GBMs containing intratumoural haemorrhage, suggesting a role for TrxR in the promotion of tumour angiogenesis and growth, but again, associations with patient survival were not presented [30]. The number of adult GBM patients included in the current study (*n* = 18) could be regarded as being rather small however the low incidence of this tumour type should be borne in mind. The standardised incidence of GBM in England is only ~5 per 100,000 persons per year [31]. The patient number in this study compares well against others that have sought to examine Trx system in GBM, i.e. the studies from Kaya et al. [30] and Kemerdere et al. [21] which included 20 and 27 GBM patients, respectively.

Molecular studies have shown that different regions of GBM tumours have different profiles of molecular abnormalities and differing expression profiles for known tumour markers [25]. This study assessed and compared the diversity of Trx system protein expression across distinct intra-tumour regions of adult GBM with results revealing that the rim region had the highest cytoplasmic TrxR expression as compared with the other two regions and that the rim and invasive margin expressed higher nuclear TrxR than the core region. Such results indicate that TrxR is overexpressed in periphery tumour regions, suggesting that such overexpression may be linked to GBM invasiveness and migration. It should be remembered, however, that this cohort is relatively small (*n* = 18) and homogeneous, and that results were obtained using a TMA, which may be not representative of the whole tissue section, especially for antigens with heterogeneous staining patterns in tumours [32]. Thus, further studies with a larger population are recommended, to evaluate the validity and reliability of using TMA to assess TrxR expression in adult GBMs, by analysing the concordance between data obtained from TMAs and whole mount sections.

The Trx system in LGG is understudied and poorly understood, with only very few studies assessing clinical importance and with none including paediatric patients. One study, investigating the prognostic significance in grade I pilocytic astrocytomas (*n* = 42), found no associations between TrxR or Trx expression and clinicopathological or survival criteria [20]. On the contrary, significant associations were seen in the current study, results showing that high cytoplasmic TrxR expression was significantly associated with tumour recurrence and adverse overall survival (*P* = 0.012), and with high nuclear TrxR expression associated with lower risk of recurrence and improved overall survival (*P* = 0.031). Such findings are similar to results obtained in a breast cancer study by our group [33], i.e. a high nuclear expression of TrxR was associated with better overall survival. The different results between cytoplasmic and nuclear TrxR may be due to the subcellular localisation of TrxR that can influence its function within the cell and therefore its involvement in the carcinogenic process and/or prediction of clinical outcome. Another study investigating 85 oligodendrogliomas showed that high Trx expression was associated with poor prognosis in univariate analysis (*P* = 0.0343) and remained significant in multivariate analysis (*P* = 0.009) [19]. This is in contrast to the current study that shows that Trx is a good prognostic factor in paediatric LGG. Differences may be due to a number of factors including study populations. The LGG cohort in the current study mainly consisted of grade I pilocytic astrocytomas from paediatric patients, whilst the study from Jarvela et al. included both grade II and III oligodendrogliomas. Besides, the different forms of Trx may also contribute to the disagreement, as the immunohistochemical technique and the antibodies that are currently available cannot discriminate the oxidised and reduced forms of the protein in archival material [34]. Apart from Trx, high TxNIP expression was also a prognostic factor for better overall survival in this cohort, which is consistent with a study conducted in locally advanced breast cancer patients [9].

Limited studies have investigated protein expression of the Trx system in HGGs with no known reports previously conducted in paediatric tumours. Haapasalo et al. [20] demonstrated that Trx expression was associated with worse prognosis in diffuse astrocytomas (*n* = 391) and Trx was an independent prognostic factor in addition to histology and proliferation. In primary human gastric cancers, high levels of Trx expression also correlated with shorter patient survival [15]. Consistent with these findings, the current study shows that cytoplasmic Trx expression associates with poor prognosis in HGGs. In a previous study of 54 glioma patients (29 LGGs and 25 HGGs), high TxNIP expression was associated with extended patient survival [22]; however, no association was observed between TxNIP and survival of HGG patients in the current study.

In the MB cohort, high expression of cytoplasmic/nuclear Trx (*P* = 0.033 and *P* = 0.007, respectively) and cytoplasmic TrxR (*P* = 0.01) significantly associated with adverse overall survival; and except for cytoplasmic Trx remained so in multivariate analysis. Such results indicate the importance of the Trx system in MB progression and the potential of using assessment of Trx system protein expression to predict clinical outcome. This is the first study to examine the expression of Trx system in MB and report on its significance in respect to patient survival. There are no previous studies investigating the role of the Trx system in MB patients or cell lines. Additional clinical studies with larger patient numbers are warranted to validate these findings.

Apart from the exploration of the relationship between protein expression and clinicopathological and survival criteria, the current study also compared levels of Trx system proteins across different brain tumour types with results revealing significant variations in expression. Interestingly MBs are more likely to show increased TrxR and Trx expression, and decreased TxNIP expression, which may confer a more aggressive behaviour. Although the adult GBMs showed some increased/decreased protein expression compared with the paediatric tumours, no significance was achieved, perhaps due to the small sample size of the adult GBM cohort (*n* = 18). A larger study, including more adult cases, could determine if variation in protein expression exists between adult and paediatric tumours.

Due to the observed difference in expression across different brain tumour types, it was of interest to see if there was an association between expression and grade. A significantly higher expression of both cytoplasmic and nuclear TrxR was observed in grade I gliomas, and grade IV gliomas had the highest TxNIP expression; whereas no significant variation in Trx expression was noted between different grades. These results are, perhaps, unexpected as previous data in gliomas often find that high levels of TrxR/Trx or low levels of TxNIP are associated with increased tumour grade. Haapasalo et al. [20] reported that grade II–IV astrocytomas (diffuse astrocytomas) showed more intense staining by TrxR and Trx than grade I astrocytomas, and within diffuse astrocytomas, TrxR and Trx showed significantly increasing expression with the malignancy grade. Esen et al. [35] found that grade I–IV astrocytomas showed significantly higher TrxR expression when compared with their normal tissue counterparts and TrxR was overexpressed according to the ascending tumour grade. Recently, Trx expression was also reported to increase with glioma grade [36]. In line with this, marked downregulation of TxNIP in HGGs was demonstrated as compared with LGGs [22]. The discordant results between the current and previous studies could be due to a number of factors including study populations, antibodies used, sample sizes, methodological problems and other potential confounding factors. It should be noted that the samples in the current study were all from paediatric patients, and the others mainly focused on adult samples. Histologically, adult and childhood brain tumours are usually similar, but they differ dramatically in their genetic and epigenetic profiles.

This current study used four independent brain tumour cohorts including both adult and paediatric patients to demonstrate associations between the expression of the Trx system and clinicopathological or survival criteria. Although some mixed results were observed between different cohorts, there was a consistent finding that high levels of cytoplasmic TrxR equated with a worse prognosis across all cohorts, supporting the prognostic value for determination of TrxR expression in different brain tumour types. The majority of the studies to date have assessed only one or maximum two members of the Trx system in focused brain tumour populations. However, the current study took a further step to examine a panel of all three members of the Trx system in a wide range of brain tumour types. Among these three members, TxNIP seemed to be the least useful marker to predict prognosis. In addition, the current study also demonstrated differential protein expression of Trx system between brain tumour types, between tumour grades and between intra-tumour regions, which perhaps indicates the complex and heterogeneous features of brain tumours and the high levels of inter-tumour and intra-tumour heterogeneity may hinder accurate diagnosis and effective treatment.

Although the present study offers several strengths, there are also some limitations. For example, for certain cohorts, some important clinical data (e.g. adjuvant treatment details) was unavailable, due to the age of certain study populations in terms of dates when patients presented i.e. non-electronic records being kept at the time with challenges facing data retrieval across national locations. Also, a proportion of cores within the TMAs could not be assessed due to missing tissue, cancer necrosis, or insufficient cancer cells, which reduced the number of cases that could be used for statistical analyses. In addition, the sample size of the cohorts, particularly the adult GBM cohort, was relatively small (*n* = 18), but even so, with the interesting results obtained, further investigation of these observations in larger cohorts are warranted. Although MB is historically considered as a single disease, it is now widely accepted that it comprises 4 distinct subgroups (i.e. WNT, SHH, group 3 and group 4) that have prognostic significance, based on their DNA methylation profiling. Unfortunately, such methylation information is not included in this study as advances in molecular testing, to allow sub-grouping, were not available at the time of presentation. In addition, the relatively small sample size (*n* = 114) might be problematic if subgrouping was conducted. Based upon the interesting data obtained, further studies are warranted, with larger numbers of patients (more contemporary, with molecular characterisation and with full treatment data), to allow subgroup analysis to be conducted.

In conclusion, this study shows that Trx system proteins, namely TrxR, Trx and TxNIP, are widely expressed across a variety of brain tumour types with high cytoplasmic TrxR expression consistently associating with worse prognosis in all brain tumour types, suggesting that TrxR is a potentially important therapeutic target in brain cancers.

## Electronic Supplementary Material

ESM 1 (PDF 462 kb)

## Data Availability

The datasets supporting the conclusions of this article are included within the article and additional files.

## Abbreviations

Trx: Thioredoxin
TrxR: Thioredoxin reductase
TxNIP: Thioredoxin-interacting protein
GBM: Glioblastoma multiforme
pLGG: Paediatric low-grade glioma
pHGG: Paediatric high-grade glioma
MB: Medulloblastoma
TMA: Tissue microarray
IHC: Immunohistochemistry
IMS: Industrial methylated spirit
TBS: Tris-buffered saline
