# Supplementary material for: Thioredoxin System Protein Expression Is Associated with Poor Clinical Outcome in Adult and Paediatric Gliomas and Medulloblastomas
Source: Mol Neurobiol. 2020 May 16;57(7):2889–901. doi: 10.1007/s12035-020-01928-z (PMC7320063; doi:10.1007/s12035-020-01928-z)
Supplement: Supplementary file 1 — (PDF 462 kb) [file 12035_2020_1928_MOESM1_ESM.pdf]

Thioredoxin system protein expression is associated with poor clinical outcome in adult and paediatric gliomas and medulloblastomas.

Anqi Yao[1,], Sarah J. Storr[1,], Khaled Al-hadyan[1,4,], Ruman Rahman[2], Stuart Smith[2], Richard Grundy[2], Simon Paine[3], Stewart G. Martin[1,5]

1 University of Nottingham, Division of Cancer and Stem Cells, Nottingham Breast Cancer Research Centre, Biodiscovery Institute, University Park, Nottingham NG7 2RD, UK.

2 University of Nottingham, Children's Brain Tumour Research Centre, Biodiscovery Institute, University Park, Nottingham NG7 2RD, UK.

3 Department of Neuropathology, Nottingham University Hospitals NHS Trust, Queen's Medical Centre, Nottingham, NG7 2UH, UK

4. Current address: Radiation Biology Section, Biomedical Physics Department, King Faisal Specialist Hospital and Research Centre, Riyadh, Kingdom of Saudi Arabia

5 Corresponding author: University of Nottingham, Division of Cancer and Stem Cells, Nottingham Breast Cancer Research Centre, Biodiscovery Institute, University Park, Nottingham NG7 2RD, UK.

Telephone: +44(0)115 823 1846

Email: stewart.martin@nottingham.ac.uk.

Supplementary Table 1: Clinicopathological variables of the adult GBM cohort.

Variable	Number of Patients	Percentage (%)
Age (years)		
≤59	10	55.6
>59	8	44.4
Gender		
Male	8	44.4
Female	10	55.6
Tumour Site		
Parietal	8	44.4
Temporal	5	27.8
Occipital	1	5.6
Frontal	4	22.2
Resection status		
Complete	14	77.8
Partial	4	22.2
Radiotherapy		
60 Gy	17	94.4
< 60 Gy	1	5.6
Temozolomide		
Yes	16	88.9
No	2	11.1
Gliadel wafer		
Yes	9	50.0
No	9	50.0
IDH-1		
Wild-type	17	94.4
Mutant	1	5.6
MGMT status		
Hypomethylated	9	90.0
Hypermethylated	1	10.0
N/A	8	
Survival status		
Living	4	28.6
Deceased	14	77.8

Abbreviations: GBM, glioblastoma; IDH1, isocitrate dehydrogenase 1; MGMT, O-6-methylguanine DNA methyltransferase; N/A, not available.

Supplementary Table 2: Clinicopathological variables of the paediatric LGG and HGG cohorts.

Variable	pLGG cohort N (%)	pHGG cohort N (%)
Age (years)		
≤3	25 (23.1)	23 (20.2)
>3	83 (76.9)	91 (79.8)
ND	18	23
Gender		
Male	57 (52.3)	80 (63.0)
Female	52 (47.7)	47 (37.0)
ND	17	10
Tumour site		
Supratentorial	38 (35.2)	84 (71.8)
Infratentorial	70 (64.8)	33 (28.2)
ND	18	20
Tumour grade		
I	92 (80.7)	0 (0.0)
II	22 (19.3)	0 (0.0)
III	0 (0.0)	35 (26.7)
IV	0 (0.0)	96 (73.3)
ND	12	6
Resection status		
Complete	NA	16 (15.8)
Partial	NA	44 (43.6)
Biopsy	NA	41 (40.6)
ND	126	36
Recurrent status		
Yes	43 (40.6)	96 (88.9)
No	63 (59.4)	12 (11.1)
ND	20	29
Survival status		
Living	77 (73.3)	19 (17.3)
Deceased	28 (26.7)	91 (82.7)
ND	21	27

Abbreviations: pLGG, paediatric low-grade glioma; pHGG, paediatric high-grade glioma; ND, not determined.

Supplementary Table 3: Clinicopathological variables of the MB cohort.

Variable	Number of Patients	Percentage (%)
Age (years)		
≤3	20	18.7
>3	87	81.3
ND	7	
Gender		
Male	78	72.2
Female	30	27.8
ND	6	
Histological Classification		
Classical	57	61.3
Desmoplastic	15	16.1
Large cell/anaplastic variant	16	17.2
Extensive nodularity	1	1.1
myogenic	4	4.3
ND	21	
Resection status		
Complete	52	61.2
Partial	33	38.8
ND	29	
Recurrent status		
Yes	42	44.7
No	52	55.3
ND	20	
Metastatic stage		
M0	56	60.9
M1	6	6.5
M2	10	10.9
M3	17	18.5
M4	3	3.3
ND	22	
Survival status		
Living	44	45.4
Deceased	53	54.6
ND	17	

Abbreviations: MB, medulloblastoma; ND, not determine

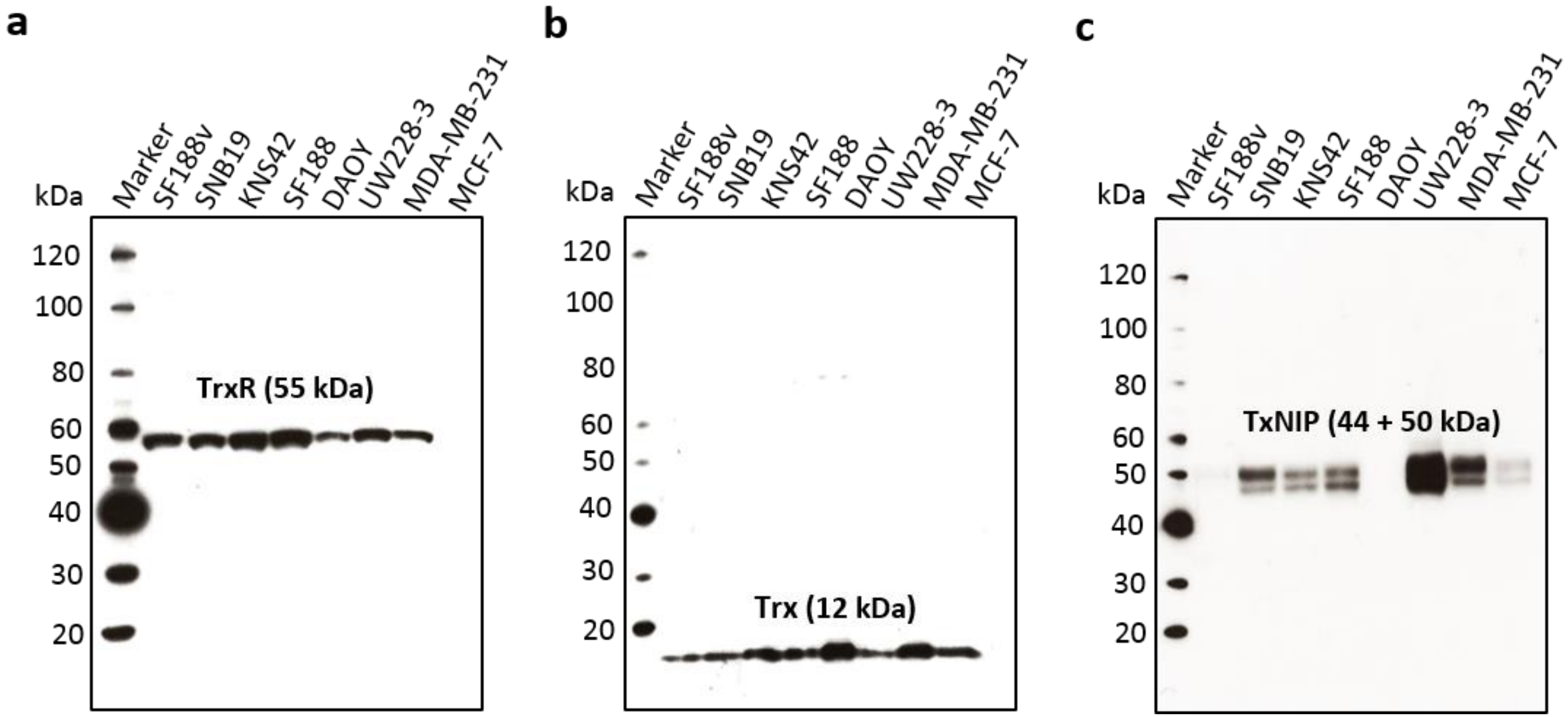

Supplementary Figure 1: The specificity of anti-TrxR, anti-Trx and anti-TxNIP antibodies. (a) TrxR, (b) Trx and (c) TxNIP expression across the panel of 6 brain (representing different tumour types) and 2 breast cancer cell lines were detected by Western blotting analysis. A single band corresponding to the molecular mass of the intended target was observed in the Western blotting studies which indicated the specificity of all antibodies. Figures present the representative blots of two independent experiments with lysates from cells with different passage numbers.

Supplementary Table 4: Intra-class correlation coefficient analysis of Trx, TrxR and TxNIP scoring across different brain tumour cohorts.

**Markers**	**Tumour types**			
	**Adult GBM**	**Paediatric LGG**	**Paediatric HGG**	**MB**
Cytoplasmic TrxR	0.701	0.724	0.702	0.712
Nuclear TrxR	0.796	0.741	0.836	0.900
Cytoplasmic Trx	0.835	0.734	0.874	0.897
Nuclear Trx	0.790	0.700	0.925	0.892
Cytoplasmic TxNIP	0.808	0.740	0.873	0.956

Abbreviations: GBM, glioblastoma; LGG, low-grade glioma; HGG, high-grade glioma; MB, medulloblastoma.

Supplementary Table 5: The median scores and ranges of TrxR, Trx and TxNIP expression in all four brain tumour cohorts.

**Markers**	**Adult GBM**			**Paediatric LGG**	**Paediatric HGG**	**MB**
	**Core**	**Rim**	**Invasive**			
Cytoplasmic TrxR	78 (30-187)	100 (77-190)	86 (50-105)	120 (5-235)	67 (0-217)	130 (0-270)
Nuclear TrxR (%)	47 (7-95)	72 (15-100)	79 (45-98)	55 (0-95)	36 (0-100)	64 (0-100)
Cytoplasmic Trx	122 (5-185)	105 (0-198)	105 (90-140)	97 (0-273)	110 (0-283)	137 (0-280)
Nuclear Trx (%)	35 (0-90)	34 (0-90)	52 (10-85)	12 (0-88)	30 (0-98)	38 (0-99)
Cytoplasmic TxNIP	175 (45-228)	140 (30-222)	170 (115-195)	173 (0-280)	188 (10-280)	150 (12-270)

Abbreviations: GBM, glioblastoma; LGG, low-grade glioma; HGG, high-grade glioma; MB, medulloblastoma.

Supplementary Table 6: X-tile cut points for stratification of TrxR, Trx and TxNIP expression in all four brain tumour cohorts.

**Markers**	**Adult GBM**			**Paediatric LGG**	**Paediatric HGG**	**MB**
	**Core**	**Rim**	**Invasive**			
Cytoplasmic TrxR	78	100	86	167	160	180
Nuclear TrxR (%)	63	80	85	77	58	58
Cytoplasmic Trx	143	150	105	27	160	172
Nuclear Trx (%)	38	23	60	14	57	60
Cytoplasmic TxNIP	175	173	175	140	195	125

Abbreviations: GBM, glioblastoma; LGG, low-grade glioma; HGG, high-grade glioma; MB, medulloblastoma.

Supplementary Table 7: Associations between TrxR expression and clinicopathological variables in adult glioblastoma (Core + Invasive margin).

**Variable**	**Nuclear TrxR (Core)**			**Cytoplasmic TrxR (Invasive)**		
	**Low**	**High**	***P*-value**	**Low**	**High**	***P*-value**
Age (years)						
≤ 59	7 (50.0%)	2 (14.3%)	0.580	5 (41.7%)	3 (25.0%)	0.545
> 59	3 (21.4%)	2 (14.3%)		1 (8.3%)	3 (25.0%)	
Gender						
Male	6 (42.9%)	1 (7.1%)	0.559	0 (0.0%)	5 (41.7%)	**0.015**
Female	4 (28.6%)	3 (21.4%)		6 (50.0%)	1 (8.3%)	
Tumour site						
Parietal	7 (50.0%)	0 (0.0%)	**0.008**	1 (8.3%)	3 (25.0%)	0.096
Temporal	0 (0.0%)	3 (21.4%)		4 (33.3%)	0 (0.0%)	
Occipital	1 (7.1%)	0 (0.0%)		0 (0.0%)	1 (8.3%)	
Frontal	2 (14.3%)	1 (7.1%)		1 (8.3%)	2 (16.7%)	
Resection status						
Complete	6 (42.9%)	7 (50.0%)	1.000	2 (16.7%)	7 (58.3%)	0.236
Partial	1 (7.1%)	0 (0.0%)		2 (16.7%)	1 (8.3%)	
IDH-1 status						
Wild-type	10 (71.4%)	3 (21.4%)	0.286	5 (41.7%)	6 (50.0%)	1.000
Mutant	0 (0.0%)	1 (7.1%)		1 (8.3%)	0 (0.0%)	

The *P* values are resultant from the Pearson χ^2 test of association or Fisher's Exact test if a cell count was less than five. Significant *P* values are indicated in bold.

Supplementary Table 8: Associations between TrxR and TxNIP expression and clinicopathological variables in paediatric low-grade glioma.

Variable	Cytoplasmic TrxR			Nuclear TrxR			Cytoplasmic TxNIP		
	**Low**	**High**	***P***	**Low**	**High**	***P***	**Low**	**High**	***P***
Age									
≤3	14 (17.9%)	3 (3.8%)	0.682	9 (11.5%)	8 (10.3%)	0.072	5 (6.1%)	12 (14.6%)	0.644
>3	54 (69.2%)	7 (9.0%)		46 (59%)	15 (19.2%)		23 (28.0%)	42 (51.2%)	
Gender									
Male	31 (46.3%)	5 (7.5%)	0.437	26 (38.8%)	10 (14.9%)	0.498	15 (21.1%)	22 (31.0%)	0.474
Female	29 (43.3%)	2 (3.0%)		20 (29.9%)	11 (16.4%)		11 (15.5%)	23 (32.4%)	
Tumour site									
Supratentorial	14 (21.2%)	6 (9.1%)	**0.002**	17 (25.8%)	3 (4.5%)	0.083	5 (7.1%)	17 (24.3%)	0.125
Infratentorial	45 (68.2%)	1 (1.5%)		28 (42.4%)	18 (27.3%)		20 (28.6%)	28 (40.0%)	
Recurrence									
No	43 (66.2%)	1 (1.5%)	**0.004**	25 (38.5%)	19 (29.2%)	**0.001**	12 (17.4%)	34 (49.3%)	**0.013**
Yes	15 (23.1%)	6 (9.2%)		20 (30.8%)	1 (1.5%)		13 (18.8%)	10 (14.5%)	

The *P* values are resultant from the Pearson χ^2 test of association or Fisher's Exact test if a cell count was less than five. Significant *P* values are indicated in bold. The table does not include the number of observations where clinicopathological data or scores were not available.

Supplementary Table 9: Associations between Trx expression and clinicopathological variables in paediatric low-grade glioma.

Variable	Cytoplasmic Trx			Nuclear Trx		
	Low	High	*P*	Low	High	*P*
Age						
≤3	2 (2.6%)	15 (19.2%)	0.643	8 (10.3%)	9 (11.5%)	0.526
>3	5 (6.4)	56 (71.8%)		34 (43.6%)	27 (34.6%)	
Gender						
Male	4 (5.9%)	32 (47.1%)	1.000	17 (25.0%)	19 (27.9%)	0.316
Female	3 (4.4%)	29 (42.6%)		19 (27.9%)	13 (19.1%)	
Tumour site						
Supratentorial	2 (30.%)	19 (28.4%)	1.000	12 (17.9%)	9 (13.4%)	0.587
Infratentorial	5 (7.5%)	41 (61.2%)		23 (34.3%)	23 (34.3%)	
Recurrence						
No	2 (3.0%)	43 (65.2%)	**0.029**	22 (33.3%)	23 (34.8%)	0.324
Yes	5 (7.6%)	16 (24.2%)		13 (19.7%)	8 (12.1%)	

The *P* values are resultant from the Pearson χ^2 test of association or Fisher's Exact test if a cell count was less than five. Significant *P* values are indicated in bold. The table does not include the number of observations where clinicopathological data or scores were not available.

Supplementary Table 10: Associations between Trx and TxNIP expression and clinicopathological variables in paediatric high-grade glioma.

Variable	Cytoplasmic Trx			Nuclear Trx			Cytoplasmic TxNIP		
	Low	High	*P*	Low	High	*P*	Low	High	*P*
Age									
≤3	16 (15.0%)	2 (1.9%)	0.145	17 (15.7%)	1 (0.9%)	0.188	6 (6.3%)	11 (11.6%)	0.114
>3	62 (57.9%)	27 (25.2%)		72 (66.7%)	18 (16.7%)		44 (46.3%)	34 (35.8%)	
Gender									
Male	41 (51.3%)	9 (11.3%)	0.564	41 (51.3%)	9 (11.3%)	0.520	21 (30.0%)	25 (35.7%)	0.314
Female	23 (28.8%)	7 (8.8%)		27 (33.8%)	3 (3.8%)		14 (20.0%)	10 (14.3%)	
Histological subtypes									
AA	16 (20.8%)	2 (2.6%)	**0.008**	16 (20.8%)	2 (2.6%)	0.189	12 (17.9%)	3 (4.5%)	**0.009**
GBM	42 (54.5%)	9 (11.7%)		44 (57.1%)	7 (9.1%)		17 (25.4%)	28 (41.8%)	
AO	3 (3.9%)	5 (6.5%)		5 (6.5%)	3 (3.9%)		5 (7.5%)	2 (3.0%)	
Tumour site									
Supratentorial	52 (67.5%)	14 (18.2%)	1.000	55 (71.4%)	11 (14.3%)	1.000	25 (36.8%)	32 (47.1%)	**0.021**
Infratentorial	9 (11.7%)	2 (2.6%)		10 (13.0%)	1 (1.3%)		9 (13.2%)	2 (2.9%)	
WHO grade									
III	19 (24.7%)	6 (7.8%)	0.545	21 (27.3%)	4 (5.2%)	0.741	16 (23.9%)	5 (7.5%)	**0.005**
IV	43 (55.8%)	9 (11.7%)		45 (58.4%)	7 (9.1%)		18 (26.9%)	28 (41.8%)	
Extent of resection									
Gross total	12 (18.5%)	1 (1.5%)	0.446	13 (20.0%)	0 (0.0%)	0.141	8 (13.3%)	4 (6.7%)	0.333
Subtotal	25 (38.5%)	8 (12.3%)		25 (38.5%)	8 (12.3%)		13 (21.7%)	18 (30.0%)	
Biopsy	15 (23.1%)	4 (6.2%)		16 (24.6%)	3 (4.6%)		9 (15.0%)	8 (13.3%)	
Recurrence									
No	10 (14.5%)	0 (0.0%)	0.189	9 (13.0%)	1 (1.4%)	1.000	7 (11.3%)	3 (4.8%)	0.167
Yes	46 (66.7%)	13 (18.8%)		50 (72.5%)	9 (13.0%)		24 (38.7%)	28 (45.2%)	

The *P* values are resultant from the Pearson $\chi 2$ test of association or Fisher's Exact test if a cell count was less than five. Significant *P* values are indicated in bold. Abbreviations: AA, anaplastic astrocytoma; GBM, glioblastoma multiforme; AO, anaplastic oligodendroglioma.

Supplementary Table 11: Associations between Trx expression and clinicopathological variables in paediatric medulloblastoma.

Variable	Cytoplasmic Trx			Nuclear Trx		
	Low	High	*P*	Low	High	*P*
Age						
≤3	7 (8.8%)	5 (6.3%)	0.495	9 (11.3%)	3 (3.8%)	0.711
>3	49 (61.3%)	19 (23.8%)		54 (67.5%)	14 (17.5%)	
Gender						
Male	35 (46.7%)	15 (20.0%)	0.859	40 (53.3%)	10 (13.3%)	0.690
Female	17 (22.7%)	8 (10.7%)		19 (25.3%)	6 (8.0%)	
Histological subtypes						
Classic	28 (45.2%)	15 (24.2%)	0.149	35 (56.5%)	8 (12.9%)	**0.041**
Desmoplastic/nodular	7 (11.3%)	1 (1.6%)		7 (11.3%)	1 (1.6%)	
MB with extensive nodularity	0 (0.0%)	2 (3.2%)		0 (0.0%)	2 (3.2%)	
Large cell/ anaplastic	5 (8.1%)	2 (3.2%)		4 (6.5%)	3 (4.8%)	
Medullomyoblastoma	2 (3.2%)	0 (0.0%)		2 (3.2%)	0 (0.0%)	
Extent of resection						
Complete	26 (44.1%)	11 (18.6%)	0.559	20 (33.9%)	2 (3.4%)	0.179
Partial	17 (28.8%)	5 (8.5%)		27 (45.8%)	10 (16.9%)	
Recurrence						
Yes	18 (28.1%)	14 (21.9%)	**0.031**	21 (32.8%)	11 (17.2%)	**0.016**
No	26 (40.6%)	6 (9.4%)		29 (45.3%)	3 (4.7%)	
Metastasis						
Absence	28 (45.9%)	10 (16.4%)	0.482	31 (50.8%)	7 (11.5%)	1.000
Presence	15 (24.6%)	8 (13.1%)		19 (31.1%)	4 (6.6%)	

The *P* values are resultant from the Pearson χ^2 test of association or Fisher's Exact test if a cell count was less than five. Significant *P* values are indicated in bold.

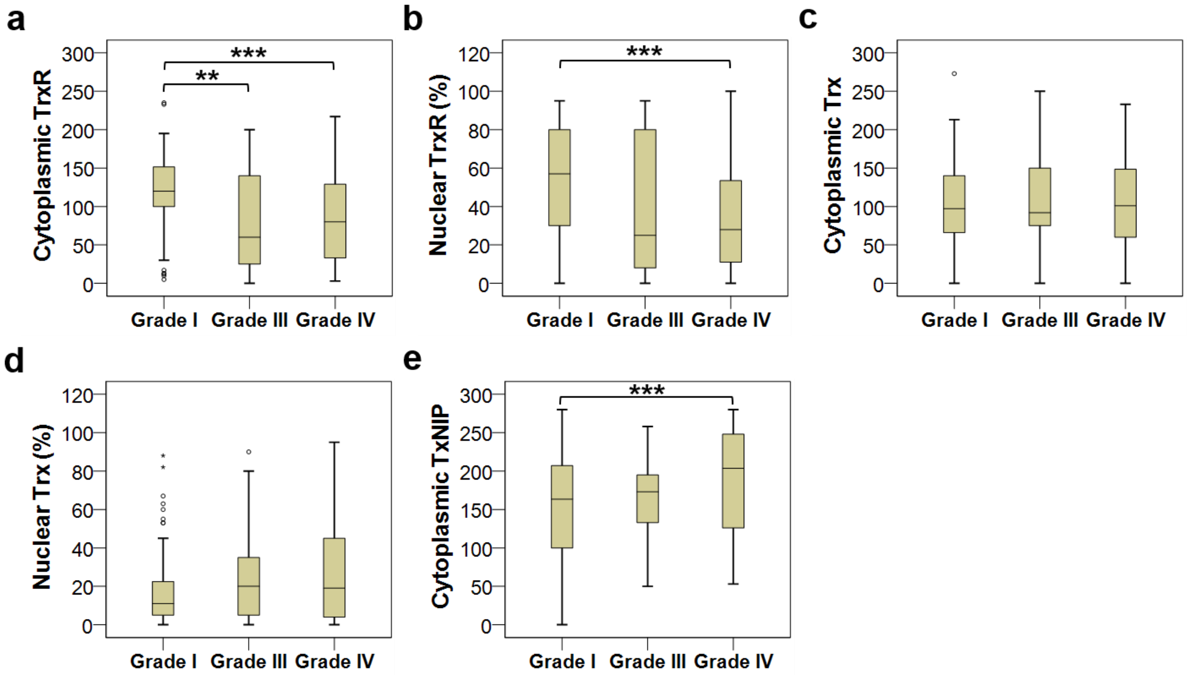

Supplementary Figure 2: Box plots of protein expression in different WHO grades of paediatric gliomas. The expression of cytoplasmic TrxR (a) is significantly higher in grade I gliomas than both grade III (P=0.010) and grade IV gliomas (P=0.001). The expression of nuclear TrxR (b) is also significantly greater in grade I gliomas than grade IV gliomas (P=0.001), but not grade III gliomas (P=0.085). No significant difference of both cytoplasmic Trx (c) (P=0.876) and nuclear Trx (d) (P=0.448) expression is detected between tumour grades. A significantly higher expression of TxNIP (e) is noted in grade IV gliomas compared to grade I gliomas (P=0.001). ** represents $P \leq 0.01$, *** represents $P \leq 0.001$.
